# Comparison of Ondansetron and Dexamethasone for Prophylaxis of Postoperative Nausea and Vomiting in Patients Undergoing Laparoscopic Surgeries: A Meta-Analysis of Randomized Controlled Trials

**DOI:** 10.1155/2016/7089454

**Published:** 2016-03-27

**Authors:** Souvik Maitra, Anirban Som, Dalim K. Baidya, Sulagna Bhattacharjee

**Affiliations:** Department of Anaesthesiology, Pain Medicine & Critical Care, All India Institute of Medical Sciences, New Delhi 110029, India

## Abstract

*Background.* Postoperative nausea and vomiting (PONV) is a significant complication after laparoscopic surgeries. Ondansetron and dexamethasone are most commonly used drugs for PONV prophylaxis. Comparisons of these two drugs have not been systematically reviewed till date.* Methods.* PubMed, PubMed Central, and CENTRAL databases were searched with the following words: “dexamethasone,” “ondansetron,” “laparoscopy,” and “PONV” to identify randomized trials that compared ondansetron and dexamethasone for PONV prophylaxis after laparoscopic surgeries.* Results.* Data of 592 patients from 7 RCTs have been included in this meta-analysis. Incidence of postoperative nausea at 4–6 h is significantly lower when dexamethasone was used instead of ondansetron (*p* = 0.04; OR 0.49, 95% CI 0.24–0.98, M-H fixed). Incidence of nausea is similar at 24 hours (*p* = 0.08, OR 0.71, 95% CI 0.48, 1.05; M-H fixed); vomiting is also similar at 4–6 h (*p* = 0.43, OR 1.27, 95% CI 0.70–2.27; M-H fixed) and also at 24 h (*p* = 0.46, OR 0.92, 95% CI 0.73, 1.16; M-H fixed).* Conclusion.* Dexamethasone is superior to ondansetron in preventing postoperative nausea after 4–6 h of laparoscopic surgeries. However, both the drugs are of equal efficacy in preventing postoperative vomiting up to 24 h after surgery. However, results should be interpreted with caution due to clinical heterogeneity in the included studies.

## 1. Introduction

Postoperative nausea and vomiting is one of the most important causes of patients' discomfort [1]. Incidence of PONV after surgery is in the range of 20–30% [2] but it may be up to 50–70% after laparoscopic surgeries [3]. Various patients related risk factors such as female sex, nonsmoking status, history of PONV, and motion sickness have been identified as risk factors for PONV. Several anaesthesia related factors such as use of opioid and nitrous oxide and duration of general anaesthesia have been implicated as risk factors of PONV. Prevention of PONV after laparoscopic surgeries is a challenge to the perioperative physicians as it is distressing for the patients and more and more such surgeries are performed on day care basis. So, an effective prophylactic regimen is highly desirable for early home discharge.

Among the drugs that are being used for PONV prophylaxis, 5HT3 antagonists, such as ondansetron, granisetron, palonosetron, and ramosetron, and dexamethasone are the two most commonly used nowadays. However, no drug has been found to provide complete PONV prophylaxis. A number of studies have compared ondansetron with dexamethasone for PONV prophylaxis after laparoscopic surgeries. These studies are not unanimous in reporting their results and there is no consensus on which drug is better in PONV prophylaxis. However, in most of the studies, the number of patients that received study drug is relatively small in number ranging from 20 to 100 and that may be one of the reasons why statistical significance could not be found. Hence, we planned this meta-analysis of randomized control trials where ondansetron has been compared with dexamethasone for PONV prophylaxis in patients undergoing laparoscopic surgeries.

## 2. Methods

A protocol of this meta-analysis has not been registered. We followed PRISMA-P 2015 [4] (preferred reporting items for systematic review and meta-analysis protocols) guidelines in this meta-analysis.

### 2.1. Eligibility Criteria

Randomized controlled trails published in English language comparing dexamethasone with ondansetron for PONV prophylaxis in patients undergoing laparoscopic surgeries under general anaesthesia were eligible to be included in this meta-analysis. Retrospective studies, prospective observational studies, case series, and reports were not included in this meta-analysis. Multiple arm trials, where dexamethasone and ondansetron have been included in two arms, have also been included in this meta-analysis.

### 2.2. Information Sources

Full text of the RCTs included in this meta-analysis was downloaded from the electronic sources. We did not contact authors for unpublished data. We did not also search for unpublished or ongoing trials.

### 2.3. Search Strategy

Two authors (Souvik Maitra and Anirban Som) independently searched PubMed and CENTRAL (the Cochrane Collaboration's Register of Clinical Trials) for eligible controlled trials using the following search words: “dexamethasone laparoscopy,” “ondansetron laparoscopy,” “ondansetron dexamethasone laparoscopy,” “ondansetron PONV laparoscopy,” and “dexamethasone PONV laparoscopy” until January 10, 2015. The details of search strategy in PubMed have been mentioned in supplementary digital content. References from the primary search result were also manually searched for potentially eligible trials.

### 2.4. Selection Process

We included published prospective randomized controlled trial where dexamethasone has been compared with ondansetron for PONV prophylaxis in patients undergoing laparoscopic surgeries. Two independent authors (Souvik Maitra and Anirban Som) selected the eligible trials. Any disagreement between two authors was solved by discussing with a third author (Dalim K. Baidya).

### 2.5. Data Collection and Data Items

Two authors independently (Dalim K. Baidya, Sulagna Bhattacharjee) extracted all data from the eligible trials. The following data were collected from each of the studies: name of the first author, year of publication, total number patients studied, type of surgery, anaesthesia details (induction agent, use of TIVA, use of nitrous oxide, and use of opioid analgesic in postoperative period), dose and time of administration of study drug, postoperative outcome (when and how assessed), use of rescue antiemetics if any, and any reported complications. Initially, all data were tabulated in Microsoft Excel*™* spread sheet. Pooled statistical analyses were performed by Souvik Maitra.

### 2.6. Risk of Bias Assessment

The quality of eligible trials was assessed using the “risk of bias” tool within Review Manager, version 5.2.3 software (Review Manager [RevMan] Version 5.2. Copenhagen: The Nordic Cochrane Centre, The Cochrane Collaboration, 2012) by two authors working independently (Souvik Maitra and Sulagna Bhattacharjee). Random sequence generation, allocation concealment, blinding, incomplete data, and selective reporting were assessed; based on the method of the trials, each was graded “yes,” “no,” or “unclear,” which reflected a high risk of bias, low risk of bias, and uncertain bias, respectively.

### 2.7. Statistical Analysis

The primary outcome of the meta-analysis was incidence of PONV in first 24 h of surgery. The secondary outcomes were incidence of PONV in first 4–6 h after surgery, incidence of nausea at first 4–6 h and 24 h after surgery, use of antiemetics, and complications.

Statistical analysis was performed by Review Manager, version 5.2.3 software (Review Manager [RevMan] Version 5.2. Copenhagen: The Nordic Cochrane Centre, The Cochrane Collaboration, 2012).

If the values were reported as median and an interquartile range or total range of values, the median itself was used to estimate mean for samples >25. The standard deviation was estimated from the median and the low and high end of the range for samples smaller than 15, as range/4 for samples from 15 to 70, and as range/6 for samples more than 70. If only an interquartile range was available, standard deviation was estimated as interquartile range/1.35 [12].

We calculated the following: (1) the odds ratio (OR) for each dichotomous outcome at individual study level; (2) the pooled OR using the Mantel-Haenszel method; (3) mean difference for each continuous outcome at individual study level; and (4) pooled mean difference using inverse variance method. All statistical variables were calculated with 95% confidence interval (95% CI). The *Q*-test was used to analyze heterogeneity of trials. When *I* ^2^ > 50%, it was considered as heterogeneous and the Mantel-Haenszel or inverse variance random effects model was used; otherwise, the fixed effects model was used. We planned to assess publication bias using visual inspection funnel plot. Where a pooled analysis was not possible (for instance, for complications), we performed a qualitative synthesis of the reported data.

## 3. Results

**Figure 1:**
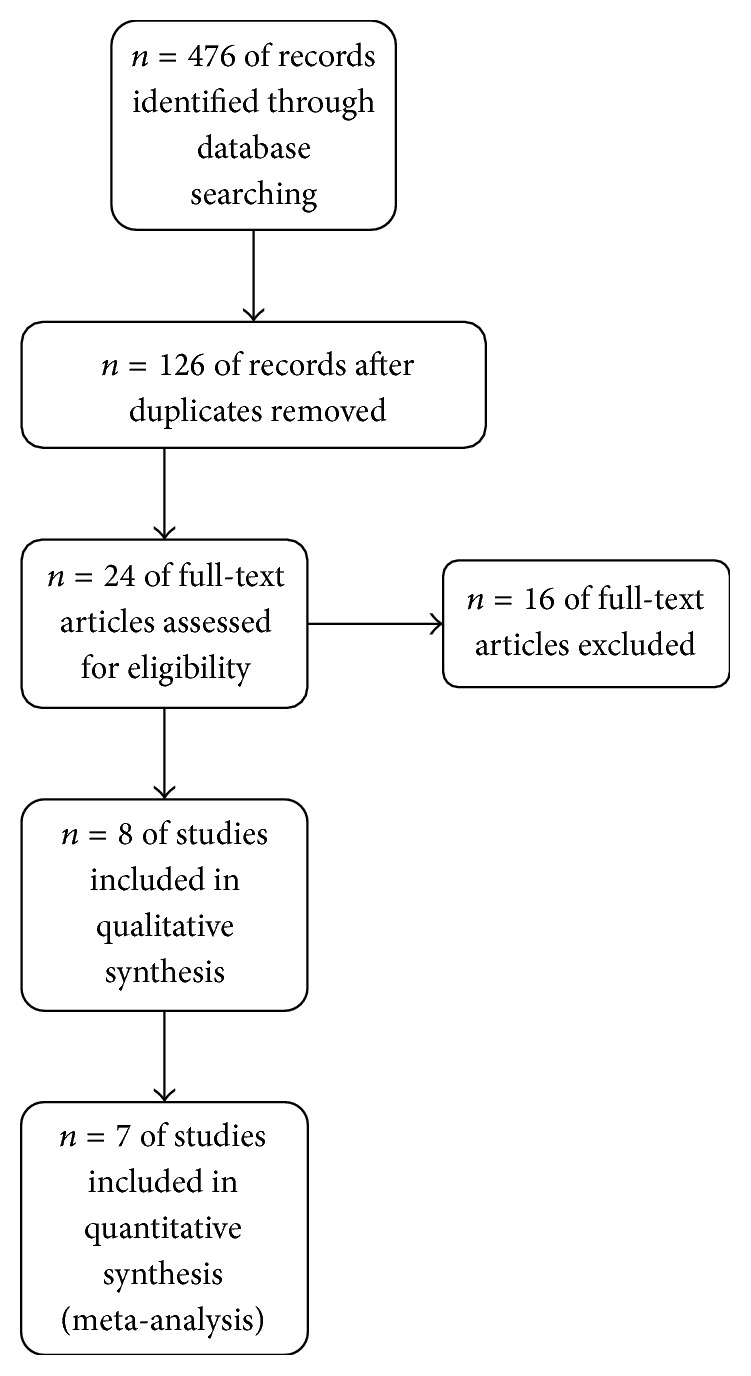
PRISMA flow diagram to show study selection procedure.

**Figure 2:**
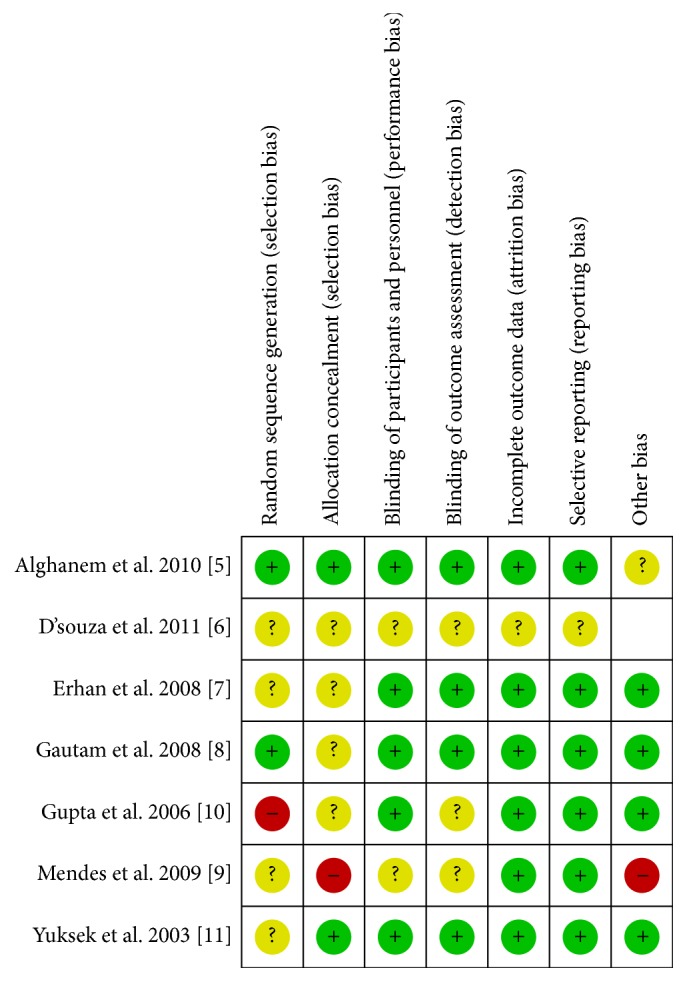
Risk of biases in the individual studies.

Initial database searching revealed 476 articles and after removing duplicate articles 126 unique articles were found. Finally eligible articles were searched from title and abstract. Eight randomized control trials fulfilled our eligibility criteria and seven of them have been included in this systematic review and meta-analysis [5–11]. One RCT [13] was not included in analysis as it reported PONV as a continuous score and a pooled analysis was not possible. Selection of the studies has been depicted in Figure 1 through a flow diagram. Characteristics of the individual trials have been summarized in Table 1. Risks of biases in the individual studies have been shown in Figure 2. No evidence of publication bias was found in any of the analyses.

### 3.1. Postoperative Nausea

**Figure 3:**
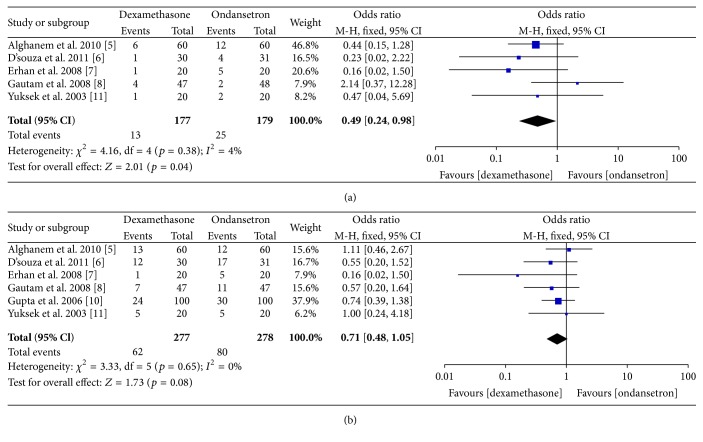
Forest plot showing odds ratio of incidence of (a) postoperative nausea at 4–6 h and (b) at 24 h at individual study level and pooled analysis level.

We have separately analyzed incidence of nausea at 4–6 postoperative hours and again within 24 hours. Incidence of postoperative nausea is significantly lower at 4–6 h when dexamethasone was used instead of ondansetron (*p* = 0.04; OR 0.49, 95% CI 0.24–0.98, M-H fixed, *n* = 356). The number of patients needed to be treated to prevent one episode of early nausea was found to be 15. However, nausea is similar at 24 hours (*p* = 0.08, OR 0.71, 95% CI 0.48, 1.05; M-H fixed; *n* = 555). Heterogeneity was insignificant in both time points. A forest plot of odds ratio at individual study level and pooled analysis level has been provided in Figure 3.

### 3.2. Postoperative Nausea and Vomiting

**Figure 4:**
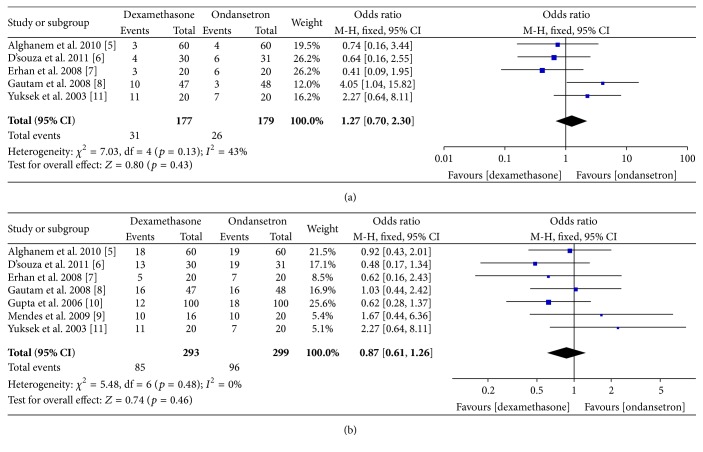
Forest plot showing incidence of (a) PONV at 4–6 h and (b) at 24 h at individual study level and pooled analysis.

Total PONV was also analyzed at 4–6 postoperative hours and 24 postoperative hours. Incidence of PONV was similar 4–6 hours (*p* = 0.43, OR 1.27, 95% CI 0.70–2.27; M-H fixed, *n* = 356) and 24 hours (*p* = 0.46, OR 0.92, 95% CI 0.73, 1.16; M-H fixed, *n* = 592). Significant statistical heterogeneity was also absent in these analyses. Forests plot of odds ratio of PONV at individual study level and pooled analysis level has been provided in Figure 4.

### 3.3. Need for Rescue Antiemetic

We have assessed rescue antiemetic use in the first 24 h after surgery. Use of rescue antiemetic is similar between two groups (*p* = 0.42; OR 0.82, 95% CI 0.50, 1.33; M-H fixed; *n* = 516).

### 3.4. Complications

None of the studies reported any significant complications attributed to either dexamethasone or ondansetron. Alghanem et al. [5] reported similar postoperative pain scores up to 24 h postoperative period. Gautam et al. [8] reported minor complications such as headache, dizziness, and urinary retention; these are found to be similar.

## 4. Discussion

Principal finding of our meta-analysis is that dexamethasone is associated with a less postoperative nausea in first 4–6 hours after laparoscopic surgeries. Postoperative vomiting and nausea at 24 hours are similar with either drug. Need for rescue antiemetic is similar with both drugs. Most important strength of our analysis is that we have not found any significant amount of heterogeneity in any analysis.

Postoperative nausea and vomiting is a common compilation after laparoscopic surgeries and may be even more distressing than postoperative pain. PONV may even delay discharge of the patients [14]. Incidence of PONV after laparoscopic cholecystectomy may be as high as 63% when no antiemetic prophylaxis is used [15]. Dexamethasone and ondansetron are the two most commonly used drugs in clinical practice for PONV prophylaxis. Individual clinical studies have found that dexamethasone is an effective antiemetic prophylaxis at a dose of 5–8 mg and recommended dose of ondansetron is 4 mg for prophylaxis [16].

Individual RCTs have found that dexamethasone and ondansetron are equally effective in PONV prophylaxis after laparoscopic surgeries. However, small sample size was the most important limitation of the RCTs that justifies importance of a meta-analysis. Interestingly we have found that dexamethasone decreases incidence of early PONV after laparoscopic surgeries and none of the previous studies has reported similar findings. Alghanem et al. [5] reported that ondansetron is less effective in preventing nausea in the 0–4 h period after surgery. However, their result did not reach statistical significance probably because of small sample size. On the contrary Gautam et al. [8] found that dexamethasone is less effective in preventing early vomiting. However, we have not found such finding in our analysis. Longer onset of action of dexamethasone may result in relative less effectiveness in preventing early PONV. Subramaniam et al. [17] found that ondansetron is more effective in preventing early PONV and dexamethasone is more effective in preventing late PONV after strabismus surgery.

These findings have not been reflected in our analysis because we believe that PONV after laparoscopic surgeries is caused by many factors such as abdominal insufflation; those may not be fully controlled by any single prophylactic drug.

Use of single dose dexamethasone is free from significant side effects including delayed wound healing [18]. Moreover, it may decrease postoperative pain after laparoscopic cholecystectomy [19]. Use of PONV prophylaxis is routine in clinical practice due to high incidence of PONV in patients who did not receive any prophylaxis. Cost of care is also an important issue in today's healthcare system. As dexamethasone is significantly cheaper than ondansetron, the former one may be a better choice for PONV prophylaxis after laparoscopic surgeries.

## 5. Limitations

Though we have not found any significant heterogeneity, different studies used different dose regimen of dexamethasone and ondansetron. However, dose ranges used in those studies are already known to be clinically effective. As PONV is multifactorial and hence anaesthetic technique may also affect incidence of PONV, possibility of biases remains there also. Numbers of included studies in this meta-analysis are small; hence, a metaregression considering TIVA, nitrous oxide, and postoperative opioid as covariate was not feasible. Surgical factors are also responsible for PONV and as included studies recruited patients from laparoscopic cholecystectomy and gynecologic laparoscopy, possibility of biases remains here also. In all studies, ondansetron is used before induction of anaesthesia; however, ondansetron is more effective when used near the end of surgery [20]; however, the reverse is true for dexamethasone [21].

## 6. Conclusion

Dexamethasone is superior to ondansetron in preventing postoperative nausea after 4–6 h of laparoscopic surgeries. Both the drugs are of equal efficacy in preventing postoperative vomiting up to 24 h after surgery. However, results should be interpreted with caution due to clinical heterogeneity in the included studies.

## Additional Points

*PubMed Search Strategy*

 (“dexamethasone” [MeSH Terms] OR “dexamethasone” [All Fields]) AND (“laparoscopy” [MeSH Terms] OR “laparoscopy” [All Fields]),
 (“ondansetron” [MeSH Terms] OR “ondansetron” [All Fields]) AND (“laparoscopy” [MeSH Terms] OR “laparoscopy” [All Fields]),
 (“ondansetron” [MeSH Terms] OR “ondansetron” [All Fields]) AND (“dexamethasone” [MeSH Terms] OR “dexamethasone” [All Fields]) AND (“laparoscopy” [MeSH Terms] OR “laparoscopy” [All Fields]),
 (“ondansetron” [MeSH Terms] OR “ondansetron” [All Fields]) AND (“postoperative nausea and vomiting” [MeSH Terms] OR (“postoperative” [All Fields] AND “nausea” [All Fields] AND “vomiting” [All Fields]) OR “postoperative nausea and vomiting” [All Fields] OR “ponv” [All Fields]) AND (“laparoscopy” [MeSH Terms] OR “laparoscopy” [All Fields]),
 (“dexamethasone” [MeSH Terms] OR “dexamethasone” [All Fields]) AND (“postoperative nausea and vomiting” [MeSH Terms] OR (“postoperative” [All Fields] AND “nausea” [All Fields] AND “vomiting” [All Fields]) OR “postoperative nausea and vomiting” [All Fields] OR “ponv” [All Fields]) AND (“laparoscopy” [MeSH Terms] OR “laparoscopy” [All Fields]).

## Figures and Tables

**Table 1:** Characteristics of individual studies.

Study	Participants	Intervention	Duration of surgery	Intraabdominal pressure	Control	Rescue antiemetic	Complications	Outcome	Source of bias
Alghanem et al. 2010 [5]	ASA 1 and ASA 2 patients aged 18–70 years scheduled for elective laparoscopic cholecystectomy	*n* = 60 patients received 8 mg dexamethasone after induction	44.5 ± 18.1 min in dexamethasone group versus 41.2 ± 13.4 in ondansetron group	10 to 16 mmHg	*n* = 60 patients received 4 mg ondansetron after induction	Metoclopramide 10 mg rescue dose, in patients with intractable nausea or lasting for at least 15 min, or at patients request anytime, or with vomiting	No significant complications	Episodes of PONV, nausea and vomiting at 0 to 4 h and 4 to 24 h intervals	

D'souza et al. 2011 [6]	Women aged 20–60 years with ASA grade I/II scheduled for gynecologic laparoscopic surgery	*n* = 31 patients received 8 mg dexamethasone before induction	Actual value not reported, no difference in surgical time	10 to 14 mmHg	*n* = 31 patients received 4 mg ondansetron after induction	Nausea/vomiting assessed with 4-point scale, retching considered as vomiting, rescue dose 10 mg metoclopramide intravenous	Not reported	Episodes of nausea and vomiting at 0–3 hours, 3–6 hours, 6–12 hours, and 12–24 hours	Use of nitrous oxide, no mention about intraoperative opioid use

Erhan et al. 2008 [7]	80 ASA I or ASA II patients (61 women and 19 men), aged 21–75 years scheduled for laparoscopic cholecystectomy	*n* = 20 patients received 8 mg dexamethasone 15 minutes before induction	72.0 ± 43.6 min in dexa group versus 69.3 ± 16.9 min ondansetron	12 mmHg	*n* = 20 patients received 4 mg ondansetron before induction	PONV recorded by nursing staff, both nausea and vomiting assessed, rescue antiemetic 10 mg metoclopramide intravenous	No significant side-effects	Incidence of nausea and vomiting was recorded during three assessment periods, 0–6 h, 6–12 h, and 12–24	

Gautam et al. 2008 [8]	150 ASA I-II patients, aged between 23 and 65 years, undergoing elective laparoscopic cholecystectomy	*n* = 50 patients received dexamethasone 8 mg before induction of anaesthesia	79.77 ± 19 min in dexa group versus 77.69 ± 19.0	Below 15 mmHg	*n* = 50 patients received 4 mg ondansetron before induction	PONV assessed by 3-point scale, 10 mg metoclopramide intravenous given when if 2 score points were reached or on patients demand	No side-effects reported	Incidence of nausea and vomiting till 24 h after surgery	

Mendes et al. 2009 [9]	77 patients aged 20 to 56 years, ASA II, BMI ≥ 35 kg·m^−2^ undergoing videolaparoscopic gastroplasty	*n* = 16 patients received 0.1 mg·kg^−1^ of dexamethasone corrected for body weight up to a maximum of 10 mg	122.5 ± 38.73 min in dexa group versus 153 ± 45.86	Not mentioned	*n* = 20 patients received 0.1 mg·kg^−1^ of ondansetron up to a maximum of 8 mg	Blinded anesthesiologist, administered 50 mg in presence of nausea or vomiting	No side effects reported	Incidence of nausea vomiting up to 24 h after surgery	Use of intraoperative morphine

Gupta et al. 2006 [10]	100 adult patients undergoing laparoscopic cholecystectomy	*n* = 100 patients received dexamethasone 5 mg 90 minutes before induction of anaesthesia	20–60 (48) min dexa group versus 20–55 (46) min in ondansetron group	12 mmHg	*n* = 100 patients received ondansetron 4 mg 90 minutes before induction of anaesthesia	Ondansetron 4 mg intravenously irrespective of the group if patients develop nausea or vomiting, administered by house staff	No postoperative complication reported	Incidence of nausea vomiting up to 48 h after surgery	

Yuksek et al. 2003 [11]	ASA PS I or II patients, aged 19–62 years and undergoing elective gynaecological laparoscopy	*n* = 20 patients received dexamethasone 8 mg	50.3 (14–98) min in dexa group versus 62.1 (23–90) min in ondansetron group	12–18 min	*n* = 20 patients received ondansetron 4 mg	Nausea vomiting assessed by 3-point ordinal scale, rescue 10 mg metoclopramide intravenous by blinded investigator	No complication was reported	Incidence of PONV immediately after surgery and at 15 min intervals for 3 h, every 30 min for 3 h, and then every 3 h for 18 h	
